# HOXD13 promotes the malignant progression of colon cancer by upregulating PTPRN2

**DOI:** 10.1002/cam4.4078

**Published:** 2021-07-17

**Authors:** Jiangyan Yin, Yi Guo

**Affiliations:** ^1^ Department of Ultrasound The First Affiliated Hospital of Chongqing Medical University Chongqing China; ^2^ Department of General Surgery Chongqing University Central Hospital (Chongqing Emergency Medical Center Chongqing China

**Keywords:** colon cancer, HOXD13, PTPRN2

## Abstract

**Purpose:**

The homeobox (HOX) family plays an important role in multi‐biological processes, such as morphogenesis and tumors. However, the function of HOXD13 in colon cancer remains unclear.

**Materials and Methods:**

The Cancer Genome Atlas database was used to analyze the expression of HOXD13 and its effect on the survival rate of colon cancer patients. Wound healing, Transwell, and clone formation were used to evaluate the effects of changes in HOXD13 expression on the function of colon cancer cells. A nude mouse xenograft tumor model was used to test the effects of HOXD13 on tumor growth in vivo.

**Results:**

Our results showed that HOXD13 was highly expressed in colon cancer and predicted a poor prognosis for patients. In in vitro experiments, the knockdown of HOXD13 can inhibit the proliferation and invasion of colon cancer cells. In vivo experiments showed the inhibited tumor growth after the knockdown of HODX13. In addition, HOXD13 bound to the protein tyrosine phosphatase receptor type N2 (PTPRN2) promoter and promoted the transcription of PTPRN2.

**Conclusion:**

We revealed the function and mechanism of HOXD13 in colon cancer and suggest that HOXD13 may be a candidate marker for the diagnosis and treatment of colon cancer.

## Graphical abstract

1. HOXD13 is highly expressed in colon cancer patients and indicates a poor prognosis.2. Both in vitro and in vivo experiments have shown HOXD13's cancer‐promoting function in colon cancer cells.3. HOXD13 binds to the PTPRN2 promoter and contributes to its expression.

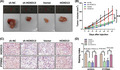

## 1 INTRODUCTION

Colorectal cancer is one of the leading causes of cancer‐related deaths. This disease ranks among the top three cancers diagnosed in men and women.^1^, ^2^ Although genetics, lifestyle, obesity, and environmental factors may be associated with the occurrence and development of colorectal cancer, the exact mechanism is not fully understood.^3^, ^4^ The treatments of colorectal cancer include endoscopic and surgical local resection, reduction of preoperative radiotherapy and chemotherapy, targeted therapy, and immunotherapy.^5^, ^6^, ^7^ Although these novel treatment methods have increased the overall survival of patients, for patients with a metastatic form of the disease, the treatment methods remain ineffective to a certain extent. Given that colorectal cancer usually presents symptoms in the late stage, the early screening and discovery of new driver genes and diagnostic markers will reduce the incidence and mortality of colorectal cancer.

Most cancers result from the gradual accumulation of genetic and epigenetic changes caused by the inactivation of tumor suppressor genes and abnormal activation of oncogenes.^8^ Typically, APC mutations trigger RAS activation or the tumor protein 53 loss of function and cause colorectal cancer development.^9^, ^10^ A large amount of accumulated evidence indicates that the homologous protein encoded by the homeobox (HOX) gene participates in epigenetic regulation during differentiation and proliferation of embryonic structures, and its abnormal expression is related to carcinogenesis and invasiveness.^11^ The HOX gene was first described as a factor related to *Drosophila* embryogenesis.^12^ In cancer, abnormal expressions of HOX genes have been found in leukemia cases.^13^, ^14^, ^15^ Several studies have also revealed the role of HOX long non‐coding RNA in the drug resistance of non‐small cell lung cancer and the progression of thyroid and liver cancers.^16^, ^17^, ^18^ In addition, the fusion of nucleoporin 98 kDa (NUP98)–HOXD13 can cause acute myeloid leukemia (AML) and T‐cell acute lymphoblastic leukemia (T‐ALL) myelodysplastic syndrome (MDS).^19^ Meanwhile, the role of HOXD13 in colorectal cancer receives limited attention.

Our study illustrates the positive regulatory role of HOXD13 in colon cancer. This research also explains the transcriptional regulatory relationship between HOXD13 and protein tyrosine phosphatase receptor type N2 (PTPRN2) and their mechanisms in the malignant progression of colon cancer. This study may enrich the role of HOX family in tumors and provide potential targets for the diagnosis and treatment of colon cancer.

## 2 MATERIALS AND METHODS

### 2.1 Cell lines and clinical specimens

Colon cancer cell lines (LoVo, HCT‐116, HCT‐8, CaCo‐2, RKO, and CW‐2) were purchased from the Cell Resource Center of the Institute of Basic Medicine, Chinese Academy of Medical Sciences. All cells were cultured in Dulbecco’s Modified Eagle Medium (DMEM) or Roswell Park Memorial Institute (RPMI) 1640 medium containing 10% fetal bovine serum (FBS) in an incubator at 37℃ with 5% CO_2_. Thirty pairs of tumors and adjacent normal specimens were immediately placed in liquid nitrogen or fixed with formalin after surgical resection. The samples stored in liquid nitrogen were used for Western blot to detect protein expression. Formalin‐fixed samples were used for immunohistochemistry (IHC). All patients gave informed consent and the approval of the ethics committee of Chongqing University Central Hospital was obtained before the experiments.

### 2.2 Cell invasion assay

Transwell experiment was used to detect cell invasion ability. Matrigel (BD, USA) was spread onto the upper chamber. After transfection and digestion into single cells, 10^5^ cells were seeded into the chamber and cultured in a serum‐free medium. Then, RPMI 1640 or DMEM containing 10% FBS was added to the lower chamber. After culturing for 20 h in an incubator containing 5% CO_2_ at 37℃, the medium was removed. The invasive cells were stained with 0.1% crystal violet for 15 min and then washed with running water. The invaded cells were observed under a microscope (Nikon, Japan).

### 2.3 Cell migration assay

Would healing experiment was used to evaluate the cell migration rate. The treated cells were seeded into 24‐well plates. After 24 h, when the cell density reached 90%, a 100 µl pipette tip was used to create a straight scratch. Then, the cells were washed with 1X phosphate‐buffered saline (PBS) to remove the suspended cells, which were then cultured in a medium containing 2% FBS and maintained in an incubator at 37℃ and 5% CO_2_. After 24 h, the cell migration rate was observed under a microscope (Nikon, Japan).

### 2.4 Colony formation

The transfected cells were seeded into a six‐well plate at 1000 cells per well and cultured in a serum‐free medium. The cells were maintained in an incubator containing 5% CO_2_ at 37℃ for 18 days. The medium was changed every 3 days. When visible clones appeared, the culture was terminated. The supernatant was discarded and the cells were washed carefully with PBS twice. Then, the cells were fixed with 4% paraformaldehyde for 15 min and then stained with 0.1% crystal violet for 20 min. Finally, the staining solution was slowly washed away with running water, and the clones were air‐dried and counted.

### 2.5 Western blot

Protein lysis (Beyotime, China; P0013C, 50 mM Tris(pH 7.4), 150 mM NaCl, 1% NP‐40, 0.5% sodium deoxycholate, and 0.1% SDS) buffer containing protease inhibitors was used to lyse tissues and cells to obtain the total protein. The harvested protein was separated in sodium dodecyl sulphate (SDS)–polyacrylamide gel electrophoresis and transferred to a polyvinylidene difluoride membrane. After blocking with 5% bovine serum albumin (BSA) for 1 h at room temperature and washing with 1 × PBS with Tween 20 (PBST) thrice, the membranes were incubated with primary antibodies (HOXD13 antibody, Abcam; ab19866, 1:500; PTPRN2 antibody, Affinity; AF9171, 1:500) for 4 h at room temperature. After washing with 1 × PBST, the membranes were incubated with horseradish peroxidase‐labeled secondary antibody at room temperature for 2 h. After washing three times with 1 × PBST, an enhanced chemiluminescence system (Millipore, USA) was used to visualize the bands following the manufacturer’s instructions. Each experiment was repeated thrice.

qRT‐PCR.

The total RNA of LoVo and CW‐2 cells was extracted with TRIzol (Sigma, USA). The quantified 2 ug total RNA was used to obtain cDNA according to the instructions of the reverse transcription kit (Takara, Japan). PCR steps for PTPRN2 RNA expression detection were as followed: 94℃, 5min; (94℃, 30 s, 56℃, 30 s, 72℃, 30 s) for 30 cycles; 72℃, 10min. GAPDH was used as an internal control. Data were quantified using a 2^−ΔΔct^ quantification method to analyze the results. The primer sequence is as follows: PTPRN2‐forward, 5′‐ACCCTGAGTCTTCCCTGTCTTC‐3′ PTPRN2‐reverse, 5′‐TTTCCTCCAGTTTGTCTTTGTTG‐3′. GAPDH‐forward, 5′‐CTCTGATTTGGTCGTATTG GG‐3′ GAPDH‐reverse, 5′‐TGGAAGAT GGTGATGGGATT‐3′.

### 2.6 IHC

After the 4 µm tissue sections were deparaffinized and dehydrated with gradient ethanol, they were placed in pH 6.0 citrate buffer and heated in a microwave oven for antigen retrieval. First, the sections were rinsed with distilled water twice, followed by rinsing with PBS twice, with each rinsing lasting for 3 min. After washing three times with 1X PBS, the tissue sections were incubated with 5% BSA to remove non‐specific binding. Then, the sections were incubated with diluted primary antibodies (HOXD13 antibody, Abcam; ab19866, 1:50; PTPRN2 antibody, Affinity; AF9171, 1:50). The sections were then placed in a humidified box at room temperature for 1 h and then stored overnight at 4℃. After discarding the primary antibodies, the diluted secondary antibody was added, and the sections were stored at 37℃ for 60 min. Then, DAB was treated for 5 min. Finally, the tissue sections were incubated with hematoxylin staining solution for 20 s and then observed under a microscope (Nikon, Japan).

### 2.7 Plasmid construction

HOXD13 expression plasmid was purchased from OriGene (RC214416, Beijing, China). PTPRN2 was purchased from Sino Biological (HG20073‐UT, Beijing, China). shRNA HOXD13 and PTPRN2 were synthesized by GENEWIZ and cloned into pENTR/U6 vector after annealing. The shRNA sequence is as follows: HOXD13‐Top, 5′‐CACCGCAGAATGCGCTCAAGTCATCCGAAGATGACTTGAGCGCATTCTGC‐3′; HOXD13‐Bottom, 5′‐GACTGCGCGCATTTCAAGTGC‐3′; PTPRN2‐Top, 5′‐CACCGGATTCAT ACCCTCCTGAAGGCGAACCTTCAGGAGGGTATGAATCC‐3′; PTPRN2‐Bottom, 5′‐AAAAGG ATTC ATACCCTCCTGAAGGTTCGCCTTCAGGAGGGTATGAATCC‐3′.

### 2.8 Xenograft tumor

A total of 24 five‐week‐old BALB/c nude mice were purchased from Charles River (Beijing, China). The mice were raised under pathogen‐free conditions and randomly divided into four groups: LoVo/sh‐NC, LoVo/sh‐HOXD13, CW‐2/pCMV6 vector, and CW‐2/HOXD13 overexpression groups. The stable cell line selected after transfection was subcutaneously injected into the mice at a concentration of 1 × 10^6^. Tumor growth was monitored by measuring the tumor diameter every 3 days. Tumor volume was calculated at the end of the study as follows: tumor volume = length × width^2^/2. After the mice were sacrificed, the tumors were removed and fixed with paraffin for further analysis. The research was approved by the Ethics Committee of Chongqing University Central Hospital and complied with the guiding principles of “Experimental Animal Experiments.”

### 2.9 Statistical analysis

All statistical analyses were performed using SPSS version 19.0 (IBM Corporation). The data are expressed as the mean and standard deviation of at least three independent experiments. Student’s *t*‐test was used to compare the significant differences between the two groups. Analysis of variance and post hoc Student–Newman–Keuls test were used to compare three or more groups. *p* < 0.05 indicates statistically significant difference with * and *p* < 0.05 indicates statistically significant difference with **.

## 3 RESULTS

### 3.1 HOXD13 is highly expressed in colon cancer and correlated with poor prognosis

**FIGURE 1:**
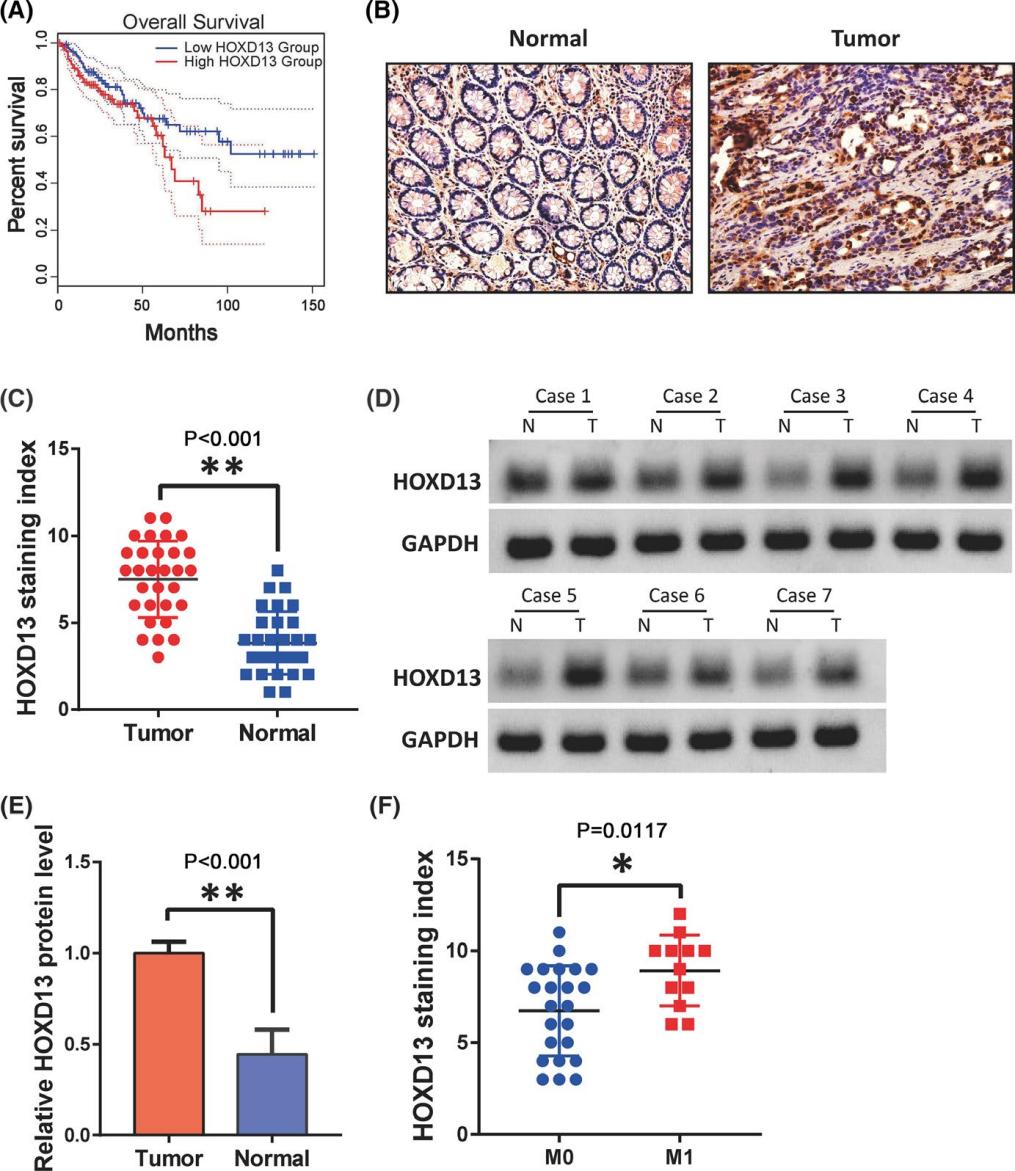
HOXD13 is highly expressed in colon cancer. (A) Survival rate of colon cancer patients with different HOXD13 expression levels. (B,C) HOXD13 levels in colon cancer and normal tissues, respectively, detected by IHC. (D,E) Western blot analysis of the protein level of HOXD13 in colon cancer and normal tissues, respectively. (F) Expression of HOXD13 in non‐metastatic and metastatic colon cancer.

We first used the Gene Expression Profiling Interactive Analysis online database (http://gepia2.cancer‐pku.cn/#index) to obtain the average of two groups of 270 colon cancer patients based on the expression of HOXD13. The results showed the prediction of a poor prognosis in the HOXD13 high‐expression group (Figure 1A). Next, we used IHC (Figure 1B,C) and Western blot (Figure 1D,E) to detect the expression of HOXD13 in clinical samples, and both showed the high expression of HOXD13 in colon cancer. The expression of HOXD13 in metastatic (M1) colon cancer is also higher than that in the non‐metastatic (M0) type (Figure 1F).

### 3.2 HOXD13 contributes to colon cancer cell proliferation, migration, and invasion

**FIGURE 2:**
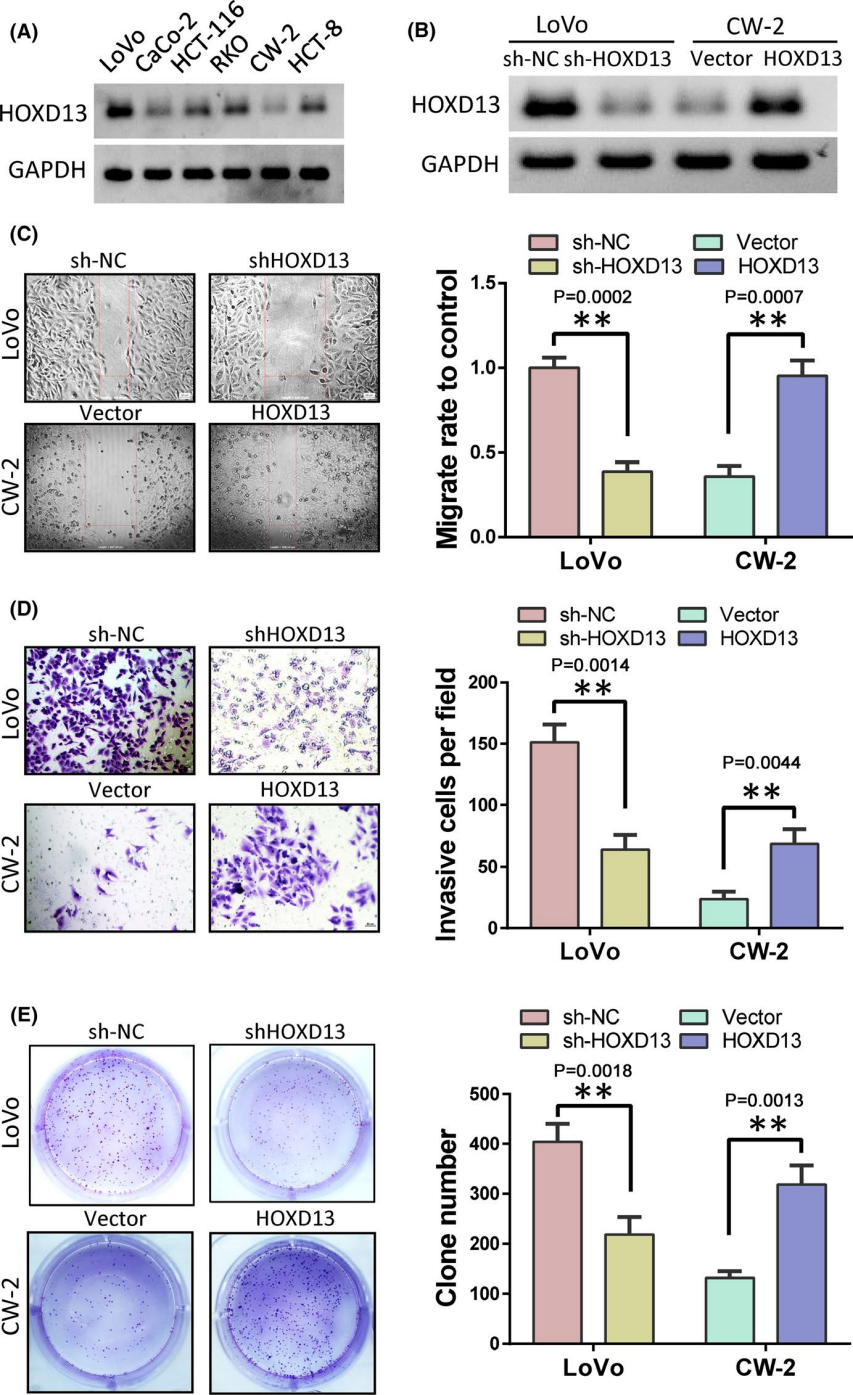
HOXD13 contributes to colon cancer cell progression. (A) HOXD13 protein levels in six colon cancer cell lines. (B) HOXD13 expression levels after the overexpression or knockdown of HOXD13. (C) Cell migration detected by wound healing. (D) Cell invasive ability determined by Transwell assay. (E). Proliferation ability of cells detected by clone formation test

After detecting the expression of HOXD13 in six colon cancer cell lines by Western blot, we selected LoVo cells with the highest expression of HOXD13 and CW‐2 cells with the lowest expression. After using shRNA to the knockdown the expression of HOXD13 in LoVo cells and overexpressing HOXD13 in CW‐2 cells, wound healing, Transwell, and colony formation experiments were performed to analyze the role of HOXD13 in colon cancer cell migration, invasion, and cloning in influencing the formation ability of cancer cells. The results showed that the knockdown of HOXD13 can inhibit the migration, invasion, and clonal formation of LoVo cells. Overexpression of HOXD13 can promote the migration, invasion, and clonal formation of CW‐2 cells. These findings show that HOXD13 is closely related to the progression of colon cancer cells (Figure 2).

### 3.3 HOXD13 promotes PTPRN2 transcription

**FIGURE 3:**
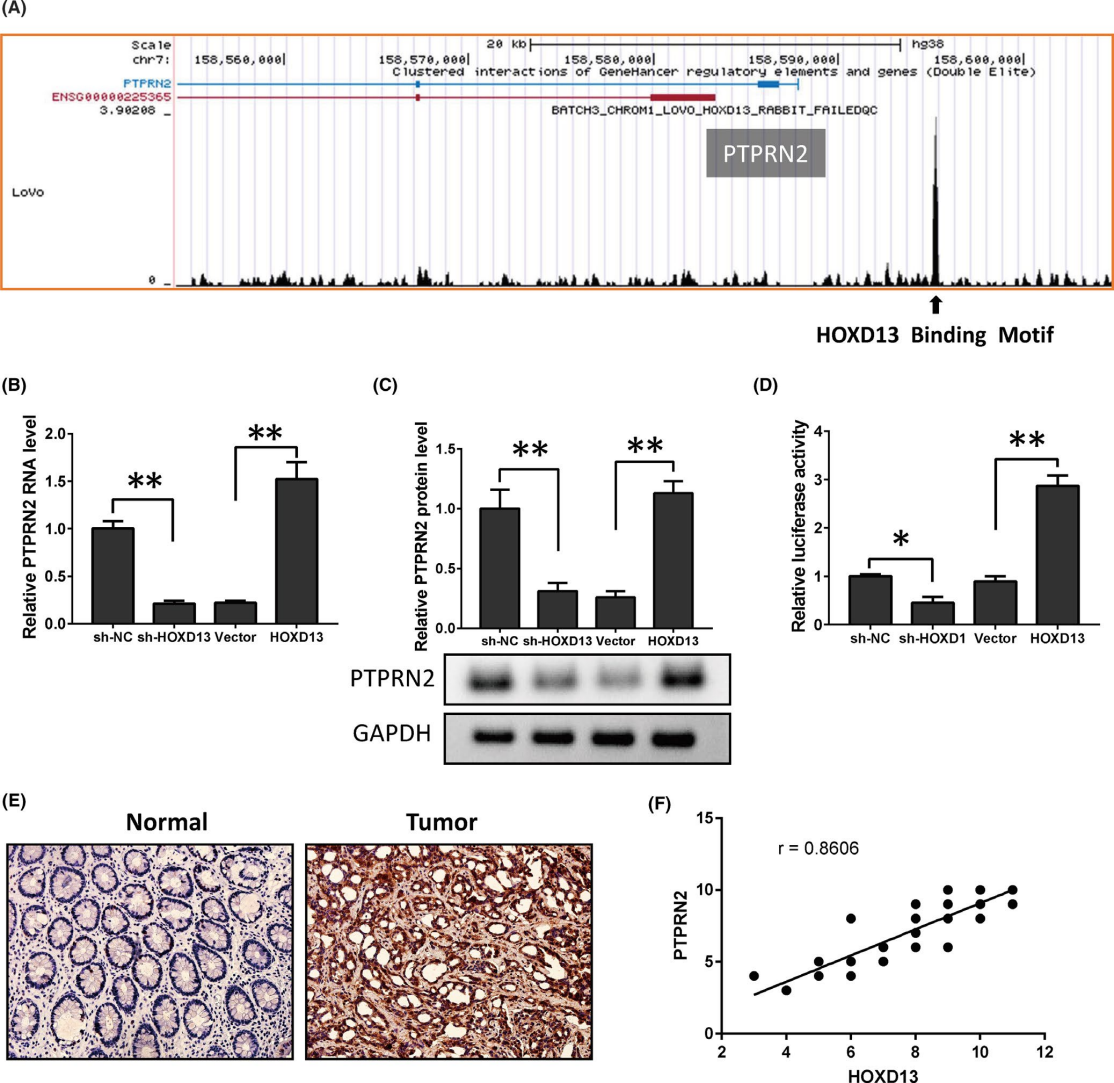
HOXD13 regulates PTPRN2 expression. (A) HOXD13 binds the PTPRN2 promoter. (B) After an exogenous change in HOXD13 expression, qRT‐PCR (B) and Western blot (C) was used to detect the protein level of PTPRN2. (D) Luciferase activity assay was used to detect whether HOXD13 binds to the PTPRN2 promoter. (E) PTPRN2 expression in colon cancer was detected by IHC. (F) Correlation was determined between HOXD13 and PTPRN2

The function of transcription factor HOXD13 in colon cancer is likely to be exerted by its regulated genes. Through Cistrome DB database (http://www.cistrome.org/db/#/) analysis, we observed that HOXD13 has a binding site in the promoter region of PTPRN2 (Figure 3A). Therefore, we detected the PTPRN2 RNA and protein after the overexpression of HOXD13 in CW‐2 cells or the knockdown of HOXD13 in LoVo cells. The results showed that the RNA (Figure 3B) and protein (Figure 3C) of PTPRN2 were upregulated after HOXD13 overexpression and decreased after HOXD13 knockdown. In addition, we cloned the promoter sequence containing the HOXD13 binding site PTPRN2 into the dual‐luciferase reporter vector. Then, we overexpressed or knocked down HOXD13 in the CW‐2 cells. The results showed that the overexpression of HOXD13 can also promote luciferase activity (Figure 3D). This result implies that HOXD13 can regulate the transcription of PTPRN2. Next, we used IHC to detect the expression of PTPRN2 in colon cancer patients and observed that the expression of PTPRN2 in the colon was higher than that in adjacent tissues (Figure 3E). Thus, the expression of PTPRN2 is positively correlated with the expression of HOXD13 (Figure 3F).

### 3.4 PTPRN2 promotes cell proliferation, migration, and invasion

**FIGURE 4:**
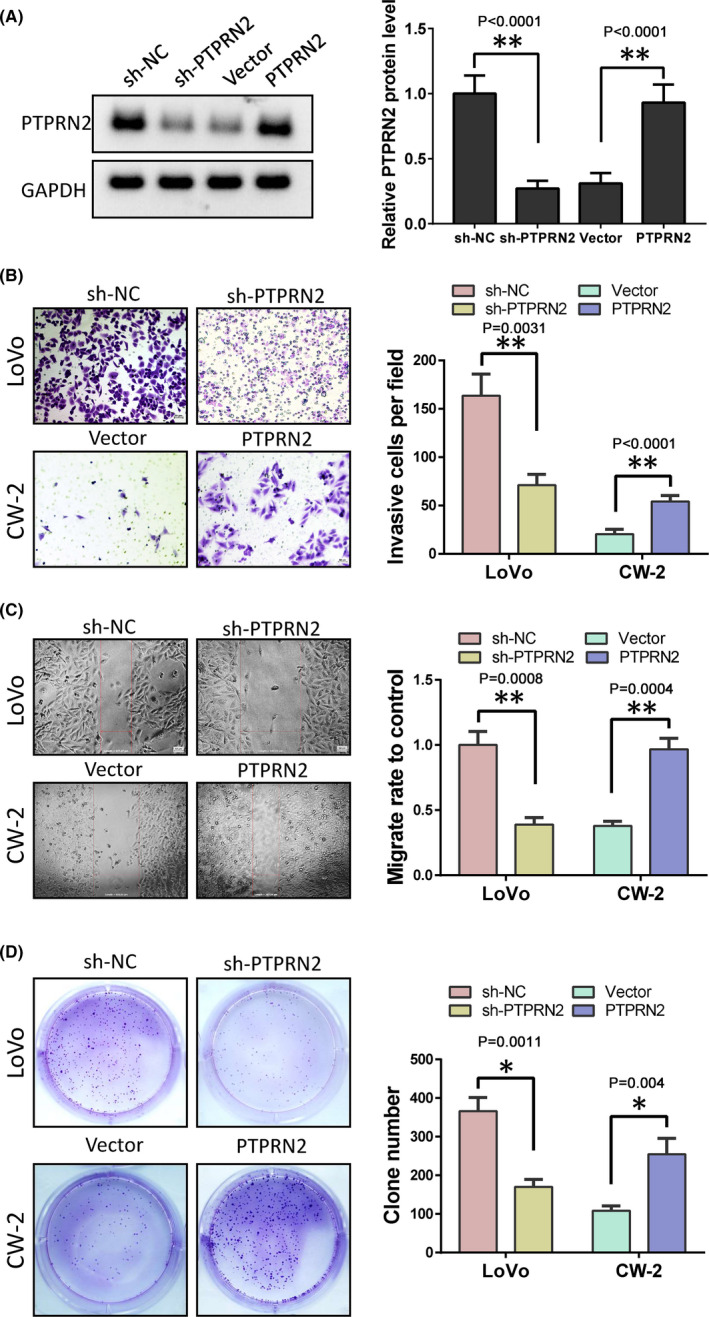
PTPRN2 promotes colon cancer cell progression. (A) Protein levels of PTPRN2 in LoVo and CW‐2 cells after treatment. (B) Cell invasive ability determined by Transwell assay analysis. (C) Cell migration detected by wound healing. (D) Proliferation ability detected by clone formation experiment

The above results indicate that the role of HOXD13 in colon cancer may be mediated by PTPRN2. Here, we first determined whether the function of PTPRN2 in colon cancer is in line with our speculation. We overexpressed HOXD13 in CW‐2 cells or knocked down PTPRN2 in LoVo cells (Figure 4A). Then, we used Transwell, wound healing, and colony formation tests to detect the effects of different levels of PTPRN2 on the phenotype of colon cancer cells. The results showed that the upregulation of PTPRN2 can promote the invasion (Figure 4B), migration (Figure 4C), and clonal formation (Figure 4D) ability of CW‐2 cells. Knockdown of PTPRN2 inhibited the above functions of LoVo cells. This result shows that the function of PTPRN2 in colon cancer is consistent with HOXD13.

### 3.5 Knockdown of PTPRN2 reverses the tumor‐promoting effect of HOXD13 in colon cancer

**FIGURE 5:**
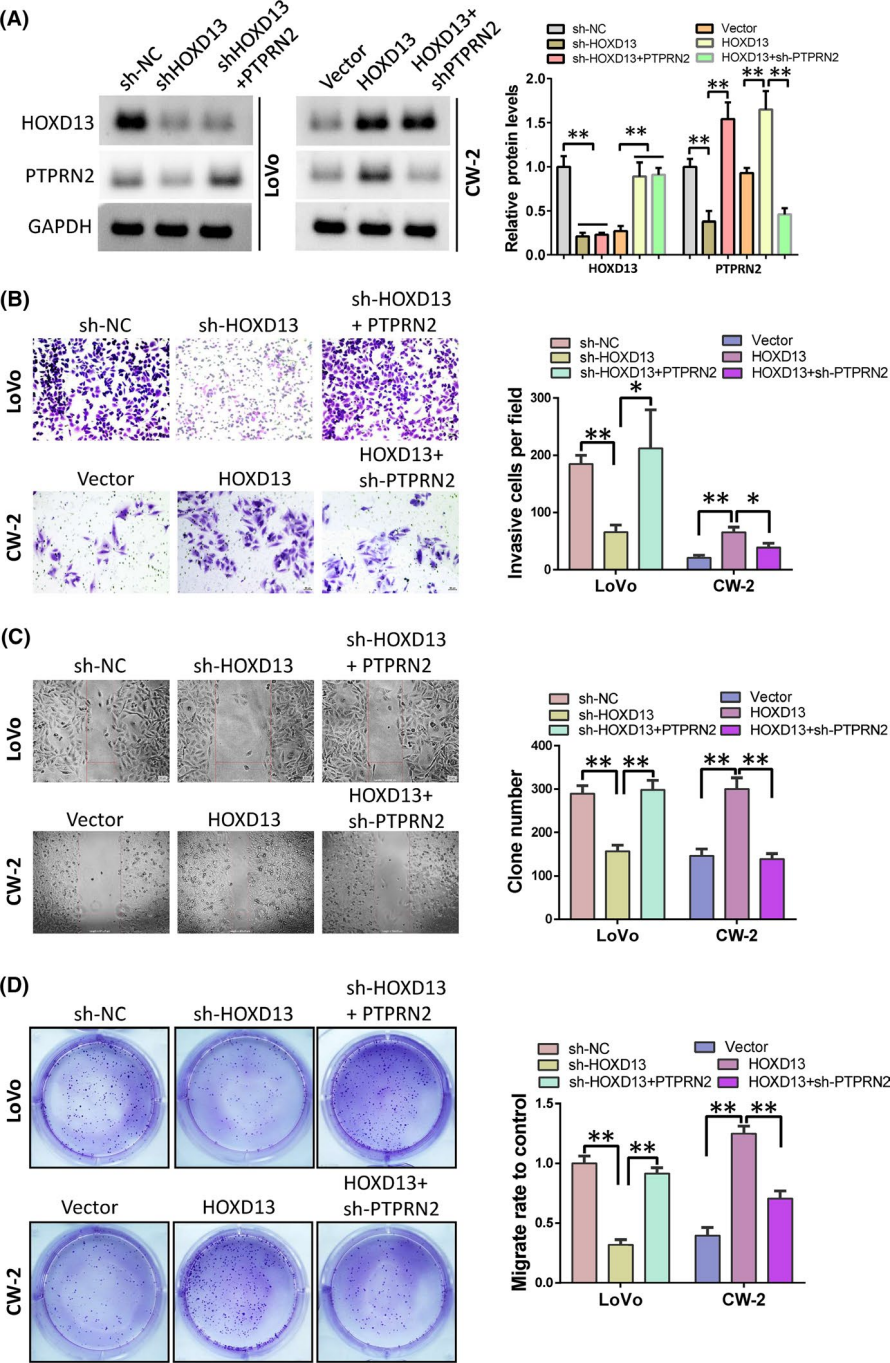
Role of HOXD13 is mediated by PTPRN2. (A) Western blot was performed to detect protein levels of HOXD13 and PTPRN2. (B) Cell invasive ability was analyzed by Transwell assay. (C) Wound healing assay was used to detect cell migration ability. (D). Colony formation was performed to analyze cell proliferation

We conducted a rescue experiment to further confirm whether PTPRN2 mediates the tumor‐promoting effect of HOXD13 in colon cancer. In LoVo cells, we overexpressed PTPRN2 and knocked down HOXD13. In CW‐2 cells, we overexpressed HOXD13 but knocked down PTPRN2 (Figure 5A). The results showed that the overexpression of PTPRN2 in LoVo cells can reverse the inhibition of cell invasion (Figure 5B), migration (Figure 5C), and clonal formation (Figure 5D) caused by the knockdown of HOXD13. The knockdown of PTPRN2 after HOXD13 overexpression in CW‐2 cells can restore the above cell functions. The findings imply that the role of HOXD13 is partially dependent on PTPRN2.

### 3.6 HOXD13 increases tumor growth in in vivo experiments

**FIGURE 6:**
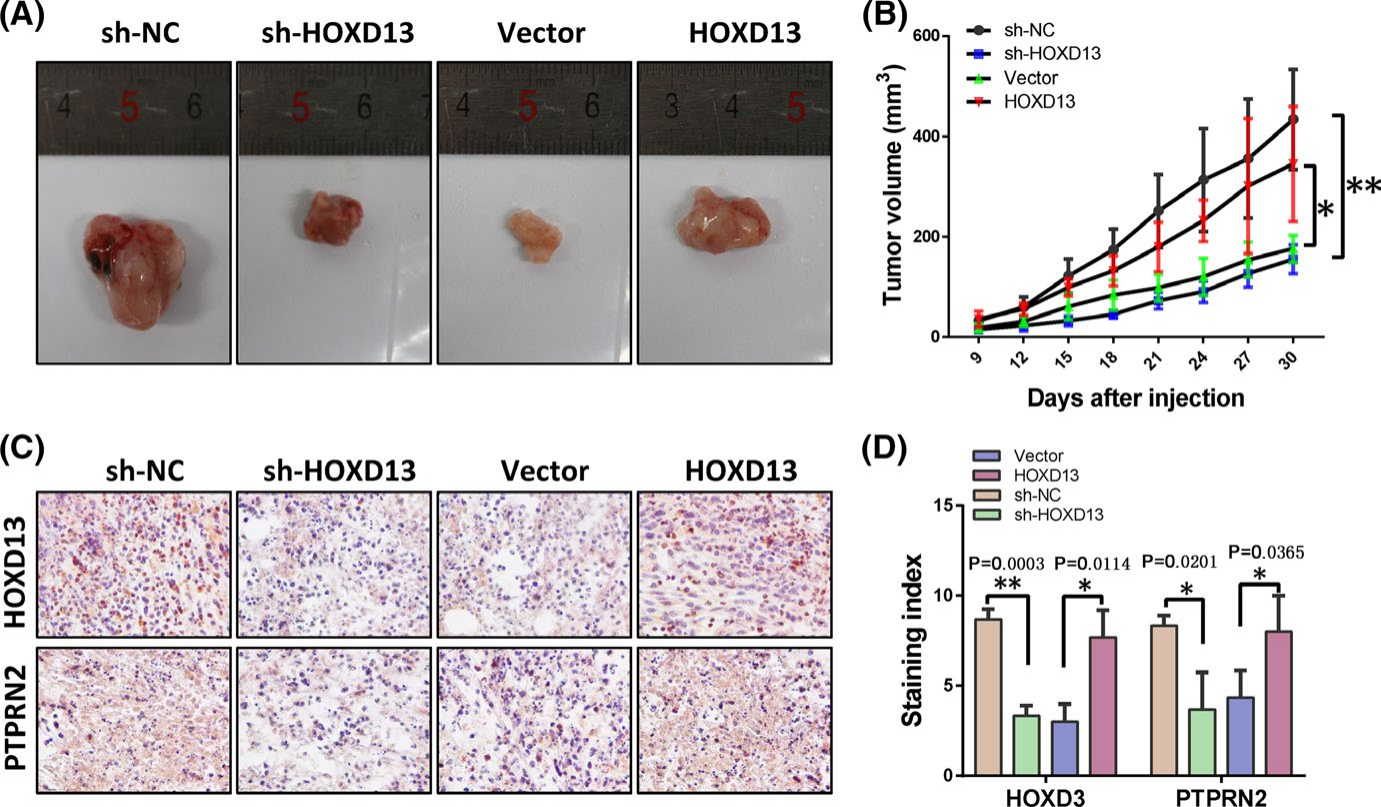
HOXD13 promotes tumor growth. (A) Solid tumors obtained by surgical resection. (B) Tumor volume of solid tumors. (C) HOXD13 and PTPRN2 expression detected by IHC. (D) Staining index of HOXD13 and PTPRN2

Clinical data and in vitro tests have shown that HODX13 promotes the progression of colon cancer through PTPRN2. To verify whether HOXD13 has the same effect in vivo, we established a stable LoVo cell line with knocked down HOXD13 and a CW‐2 stable cell line overexpressing HOXD13. These kinds of cells and their respective control cells were inoculated to BALB/c nude mice to observe the effects of HOXD13 on tumor growth. The results showed that the knockdown of HODX13 can inhibit tumor growth, whereas its overexpression can promote tumor growth (Figure 6A,B, respectively). HOXD13 and PTPRN2 in solid tumors obtained by immunohistochemical detection revealed that HOXD13 can promote the expression of PTPRN2 (Figure 6C,D).

## 4 DISCUSSION

In the process of tumor progression, cancer cells can invade surrounding tissues and penetrate the blood or lymph circulation, thereby causing the spread of tumor cells. HOXD13 belongs to the HOX family. Its main function is as a transcription factor, and it plays an important role in tissue morphogenesis and cancer progression. The NUP98–HOXD13 fusion oncogene induces thymocyte self‐renewal and promotes the development of MDS progressing to AML and T‐ALL.^19^, ^20^ Our experimental results show that HOXD13 is highly expressed in colon cancer and is positively correlated with colon cancer metastasis. The knockdown of HOXD13 can significantly inhibit the proliferation, migration, and invasion of colon cancer cells. In vivo experiments showed that the overexpression of HOX13 can promote tumor growth, whereas its knockdown results in the opposite. In addition, HOXD13 binds to the promoter of PTPRN2 and regulates the transcription of PTPRN2. The cancer‐promoting effect of HOXD13 in the colon is partly achieved through PTPRN2. However, several previous results are inconsistent with our conclusions. In breast cancer, the methylation modification and low expression of the HOXD13 promoter are positively correlated with the poor prognosis of breast cancer patients.^21^, ^22^ In addition, HOXD13 inhibits prostate cancer metastasis and epithelial‐mesenchymal transition by inhibiting SMAD1.^23^ This finding shows the plasticity of HOXD13 in the biological regulation of tumors and may be related to the properties of HOXD13 and transcription factors or cofactors. However, these findings need to be further verified by experiments.

PTPRN2 belongs to the N‐type family of PTP receptors. It is usually expressed in the nervous system and endocrine cells and participates in the regulation of insulin secretion.^24^, ^25^ PTPRN2 is overexpressed in a series of tumors, including breast and pancreatic cancers.^26^, ^27^ We discovered that PTPRN2 is highly expressed in colon cancer and predicts a poor prognosis. The knockdown of PTPRN2 expression in colon cancer cells can inhibit cell migration, invasion, and clonal formation. In addition, our results show that the promoter region of PTPRN2 has HOXD13‐binding motifs, and the transcription of PTPRN2 is regulated by HOXD13. The promotion of HOXD13 in colon cancer is partly achieved by PTPRN2. This result may suggest that the anti‐tumor targeting of PTPRN2 not only requires the inhibition of PTPRN2 at the protein level but also the abnormal transcription of PTPRN2 mediated by upstream HOXD13.

Overall, HOXD13 promotes the malignant progression of colon cancer through PTPRN2. In vitro experiments have shown that HOXD13 can promote colon cancer cell migration, invasion, and clonal formation. In vivo experiments also revealed that HOXD13 can promote tumor growth. In addition, HOXD13 expression is high in patients with colon cancer and predicts a poor prognosis. This finding illustrates the important role of HOXD13 in colon cancer and its potential as a therapeutic target.

## 5 CONFLICT OF INTEREST STATEMENT

The authors report no conflicts of interest.

## 6 ETHICS STATEMENT

All procedures involving animals were approved and performed in accordance with the ethical standards of the Institutional Animal Care and Use Committee at Chongqing University Central Hospital and complied with the guiding principles of “Experimental Animal Experiments.”

## AUTHOR CONTRIBUTIONS

Jiangyan Yin responsible for experiments and data analysis, Yi Guo conceived and designed the study.

## Data Availability

The datasets used during the current study are available from the corresponding author on reasonable request.
